# Supplementary material for: Tumor Necrosis with Adjunction of Preoperative Monocyte-to-Lymphocyte Ratio as a New Risk Stratification Marker Can Independently Predict Poor Outcomes in Upper Tract Urothelial Carcinoma
Source: J Clin Med. 2021 Jul 3;10(13):2983. doi: 10.3390/jcm10132983 (PMC8267944; doi:10.3390/jcm10132983)

Figure S1 ROC analysis for optimal cut-off value of MLR according to cancer-specific mortality of UTUC.

ROC: receiver operating characteristic; MLR: monocyte-to-lymphocyte ratio; UTUC: upper tract urothelial carcinoma; AUC: area under curve.

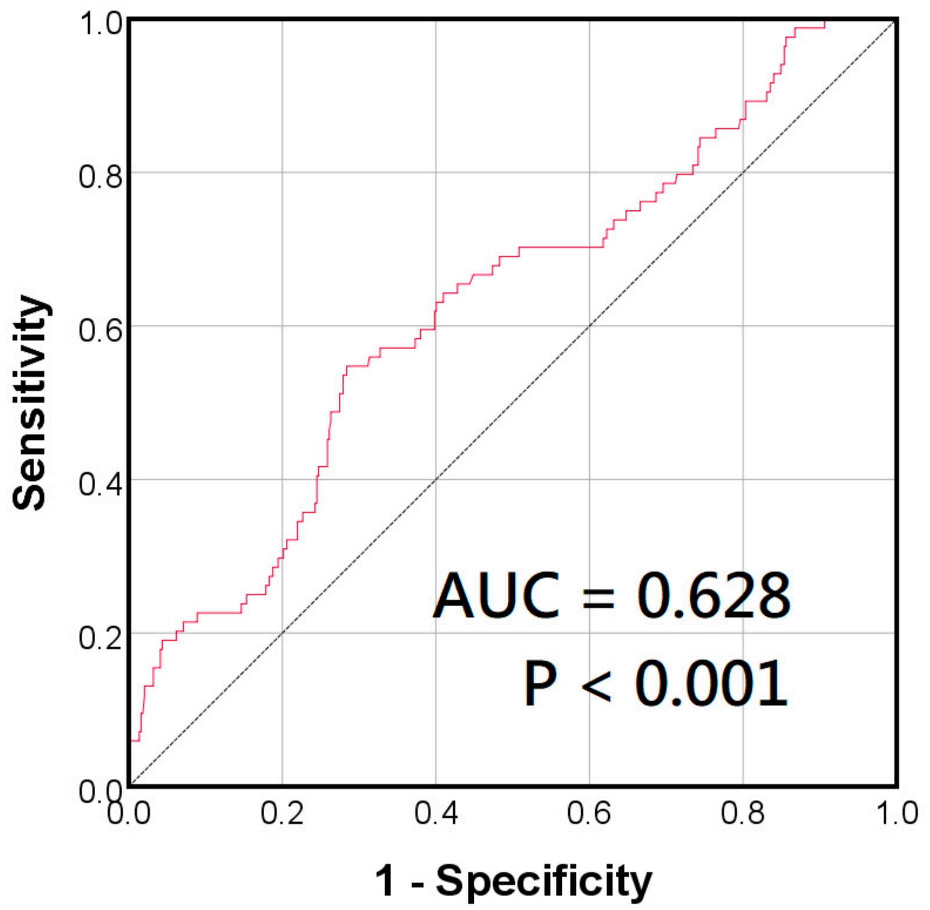

Figure S2 Comparison of preoperative MLR values in UTUC patients without versus with tumor necrosis.

UTUC: upper tract urothelial carcinoma; MLR: Monocyte-to-lymphocyte ratio.

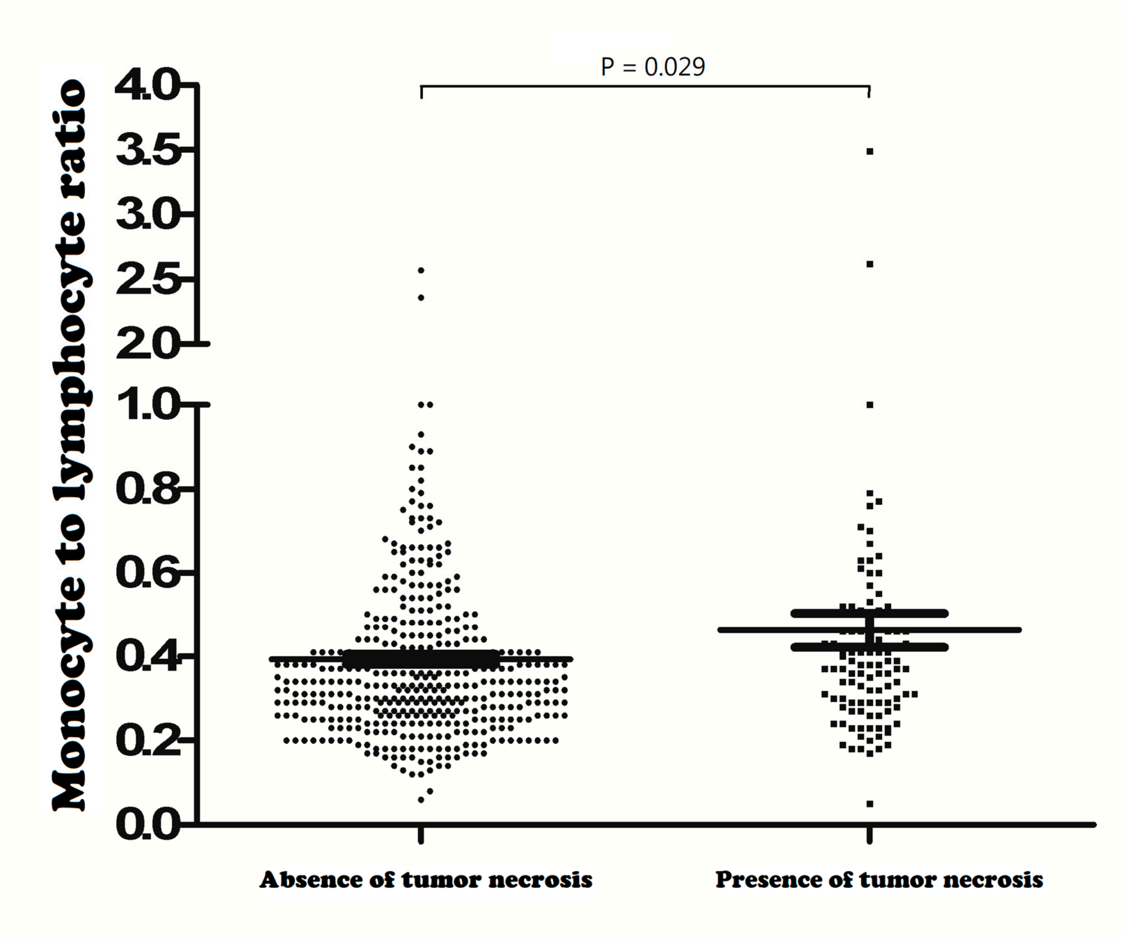

Figure S3 Kaplan-Meier survival curves for overall survival (A), cancer-specific survival (B), and recurrence-free survival (C) in UTUC patients according to preoperative MLR (high-level versus low-level). UTUC: upper tract urothelial carcinoma; MLR: monocyte-to-lymphocyte ratio.

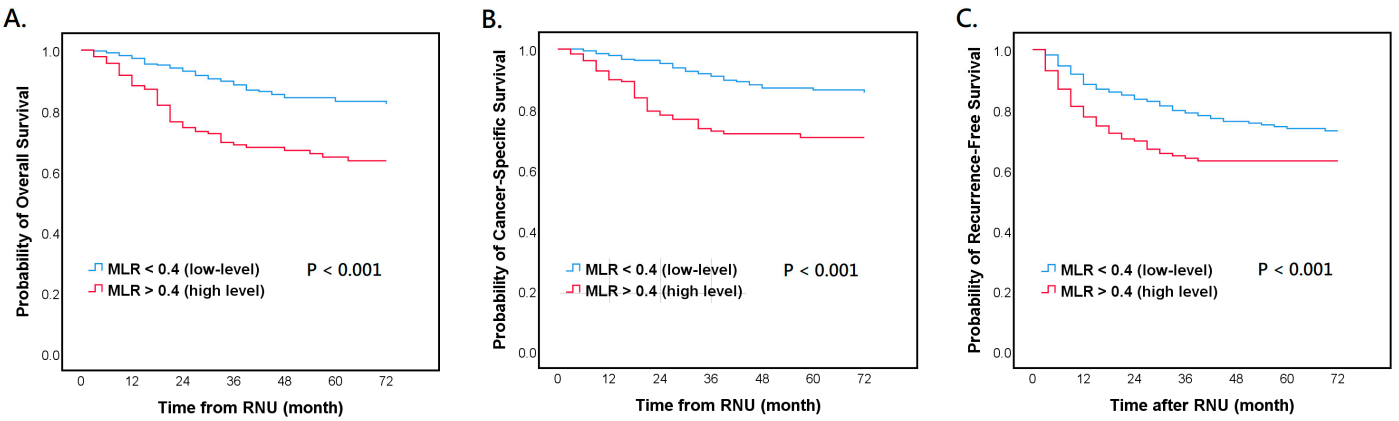

Supplement: Supplementary file 1 [file jcm-10-02983-s001.zip › jcm-1226766-supplementary.pdf]
